# Crude Oil Price Forecasting Based on Hybridizing Wavelet Multiple Linear Regression Model, Particle Swarm Optimization Techniques, and Principal Component Analysis

**DOI:** 10.1155/2014/854520

**Published:** 2014-05-08

**Authors:** Ani Shabri, Ruhaidah Samsudin

**Affiliations:** ^1^Department of Science Mathematic, Faculty of Science, Universiti Teknologi Malaysia, 81310 Johor, Malaysia; ^2^Department of Software Engineering, Faculty of Computing, Universiti Teknologi Malaysia (UTM), 81310 Johor, Malaysia

## Abstract

Crude oil prices do play significant role in the global economy and are a key input into option pricing formulas, portfolio allocation, and risk measurement. In this paper, a hybrid model integrating wavelet and multiple linear regressions (MLR) is proposed for crude oil price forecasting. In this model, Mallat wavelet transform is first selected to decompose an original time series into several subseries with different scale. Then, the principal component analysis (PCA) is used in processing subseries data in MLR for crude oil price forecasting. The particle swarm optimization (PSO) is used to adopt the optimal parameters of the MLR model. To assess the effectiveness of this model, daily crude oil market, West Texas Intermediate (WTI), has been used as the case study. Time series prediction capability performance of the WMLR model is compared with the MLR, ARIMA, and GARCH models using various statistics measures. The experimental results show that the proposed model outperforms the individual models in forecasting of the crude oil prices series.

## 1. Introduction

Crude oil prices do play significant role in the global economy and constitute an important factor affecting government's plans and commercial sectors. Forecasting crude oil price is among the most important issues facing energy economists. Therefore, proactive knowledge of its future fluctuations can lead to better decisions in several managerial levels.

The literature dealing with forecasting crude oil is substantial. The application of the classical time series models such as autoregressive moving average (ARMA) (Yu et al. [1], Mohammadi and Su [2], and Ahmad [3]) and econometric model such as generalized autoregressive conditional heteroscedasticity (GARCH) type models (Agnolucci [4], Wei et al. [5], Liu and Wan [6]) for crude oil forecasting has received much attention in the last decade. But because the crude oil price has the volatility, nonlinearity, and irregularity, the classical and econometric model can lead to the decrease of the accuracy.

Due to the limitations of the classical and econometric models, soft-computing models, such as neural fuzzy (Ghaffari and Zare [7]), artificial neural networks (Kaboudan [8], Mirmirani and Li [9], Shambora and Rossiter [10], and Yu et al. [11]), support vector machines (Xie et al. [12]), and genetic programming (GP), provide powerful solutions to nonlinear crude oil price prediction. Many experiments found that the soft-computing models often had some advantages over statistical-based models. However, these AI models also have their own shortcomings and disadvantages. For example, ANN often suffers from local minima and over-fitting, while other soft-computing models, such as SVM and GP, including ANN, are sensitive to parameter selection [1].

To remedy the above shortcomings, some hybrid methods have been used recently to predict crude oil price and obtain the best performances. In last year, wavelet transform has become a useful method for analyzing such as variations, periodicities, and trends in time series. The hybrid models with wavelet transform processes have been improved for forecasting. For example wavelet-neural network (Jammazi and Aloui [13], Qunli et al. [14], and Yousefi et al. [15]), wavelet-least square support vector machines (LSVM) (Bao et al. [16]), and wavelet-fuzzy neural network (Liu et al. [17]) have been employed recently on some studies in crude oil forecasting. They observed that the wavelet transform fairly improves forecasting accuracy.

A major drawback of wavelet transform for direction prediction is that the input variables lie in a high-dimensional feature space depends on the number of sub-time series components. Because the number of sub-time series components for wavelet is inadvisable to be too many, in this study principal component analysis (PCA) is proposed to reduce the dimensions of sub-time series components.

The multiple linear regressions (MLR) model that is much easier to interpret is considered as an alternative to ANN model. In this paper, a hybrid wavelet multiple linear regression (WMLR) model integrating wavelet and MLR is proposed for short-term daily crude oil price forecasting. The study applies particle swarm optimization (PSO) to adopt the optimal parameters to construct the MLR model. For verification purpose, the West Texas Intermediate (WTI) crude oil sport price is used to test the effectiveness of the proposed WMLR ensemble learning methodology. Finally to evaluate the model ability, the proposed model was compared with individual ARIMA and GARCH models.

## 2. Methodology 

### 2.1. The ARIMA Model

The most comprehensive of all popular and widely known statistical methods used for time series forecasting are Box-Jenkins models (Box and Jenkins [18]). It has achieved great success in both academic research and industrial applications during the last three decades. The general form of ARIMA models can be expressed as

(1) $$\begin{array}{c} y_{t}=\overset{p}{\underset{i=1}{\sum }}\phi_{i}y_{t-i}+\overset{q}{\underset{i=1}{\sum }}\theta_{i}e_{t-i}+e_{t}, \end{array}$$

where *p* is the order of the autoregressive, *q* is the order of the moving average, and *e*_*t*_ is the random error. The Box-Jenkins methodology is basically divided into four steps: identification, estimation, diagnostic checking, and forecasting.

### 2.2. The GARCH Model

GARCH models have found extensive application in the literature and the most popular volatility model is GARCH (1,1) model proposed by Bollerslev [19]. The standard GARCH (1,1) can be described as follows:

(2) $$\begin{array}{c} r_{t}=\mu_{t}+\varepsilon_{t}=\mu_{t}+h_{t}^{1/2}\eta_{t}\;\eta_{t}\sim N\left(0,1\right),\\ h_{t}=\omega +\alpha \varepsilon_{t-1}^{2}+\beta h_{t-1}, \end{array}$$

where *μ*_*t*_ denote the conditional mean and *h*_*t*_ is the conditional variances and *η*_*t*_ is a standardized error and *r*_*t*_ = ln⁡(*x*_*t*_/*x*_*t*−1_) is log return.

### 2.3. Multiple Linear Regressions

Multiple linear regressions (MLR) model is one of the modelling techniques to investigate the relationship between a dependent variable and several independent variables. Let the MLR have *p* independent variables with *n* observations. Thus the MLR can be written as

(3) $$\begin{array}{c} Y=w_{0}+w_{1}x_{i}+w_{2}x_{2}+\cdots +w_{p}x_{p}+\varepsilon_{t}, \end{array}$$

where *w* are regression coefficients, *Y* is dependent variable, *x*_*i*_ are independent varaiables and *ε*_*t*_ is fitting errors. The method of least squares is generally used to estimate the coefficients model. In many applications, the results of a least squares fit are often unacceptable when the model is wrong or when the model is misspecified (Bozdogan and Howe [20]).

In this study, particle swarm optimization (PSO) method is presented to determine the optimal parameters of the MLR model. The PSO methods have proven to be very effective in solving a variety of difficult global optimization problems in forecasting (Chen and Kao [21] and Alwee et al. [22]), heat problem (Ma et al. [23] and Tyagi and Pandit [24]), and dynamic environments (Liu et al. [25]).

The classic solution of MLR model involves the minimization of the sum of the square errors between the model-predicted value and the corresponding data value:

(4) $$\begin{array}{c} \min f\left(w\right)=\overset{n}{\underset{i=1}{\sum }}{\left(Y_{i}-{\hat{Y}}_{i}\right)}^{2}, \end{array}$$

where *n* is the number of training data samples, *Y*_*i*_ is the actual value, and ${\hat{Y}}_{i}$ is the forecasted value of train data. The same methodology was used to solve this problem using PSO algorithms. The solution with a smaller fitness *f*(*w*) of the training data set has a better chance of surviving in the successive generations.

### 2.4. Particle Swarm Optimization

Particle swarm optimization (PSO) is a population-based heuristic method inspired by the collective motion of biological organisms, such as bird flocking and fish schooling, to simulate the seeking behavior to a food source (Bratton and Kennedy [26]). The population of PSO is called a swarm and each individual in the population of PSO is called a particle. The PSO begins with a random population and searchers for fitness optimum just like genetic algorithm (GA). To find the optimum solution, each particle adjusts the direction through the best experience which it has found (*p*_best_) and the best experience that has been found by all other members (*g*_best_). Therefore, the particles fly around in a multidimensional space towards the better area over the search process.

Each particle consists of three vectors: the position for *i*th individual particle can be denoted as *X*_*i*_ = (*x*_*i*_^(1)^, *x*_*i*_^(2)^,…, *x*_*i*_^(*D*)^), the best previous position *p*_best_ that the *i*th particle has searched is *P*_*i*_ = (*p*_*i*_^(1)^, *p*_*i*_^(2)^,…, *p*_*i*_^(*D*)^), and the fly velocity of the *i*th is *V*_*i*_ = (*v*_*i*_^(1)^, *v*_*i*_^(2)^,…, *v*_*i*_^(*D*)^). The performance of each particle is measured using a fitness function varying from problem in hand. During the iterative procedure, the *i*th particle at iteration *t* is updated by

**Figure 1:**
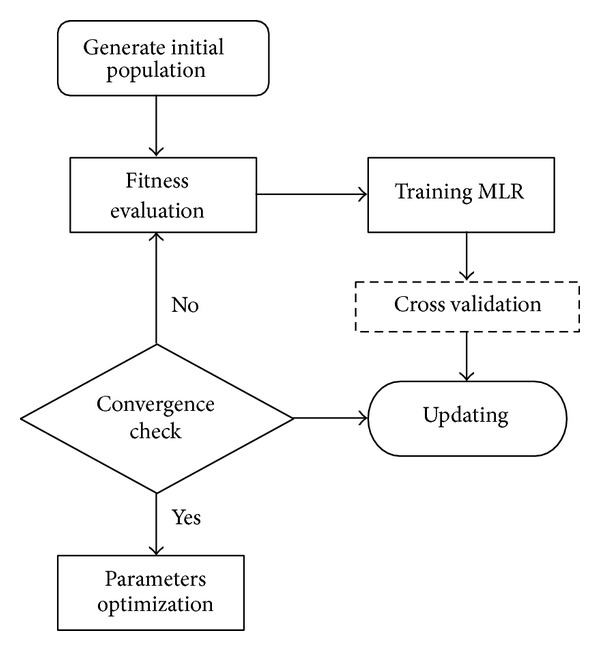
Flowchart of PSO algorithm.

(5) vid(t+1)=ω′×vid(t)+c1×φ1×[pid(t)−xid(t)]+c2×φ2×[pgd(t)−xid(t)],xid(t+1)=xid(t)+vid(t+1),

where *ω* is called inertia weight, *c*_1_ and *c*_2_ are acceleration constants, and *φ*_1_ and *φ*_2_ are stochastic value of [0, 1]. In a PSO system, particles change their positions at each time step until a relatively unchanging position has been encountered or a maximum number of iterations have been met. In general, the performance of each particle is measured according to a fitness function, which is problem dependent. In MLR model, (4) is the fitness function under consideration.  Figure 1 shows the flowchart of the developed PSO algorithm. For further details regarding PSO, please refer to Kennedy and Eberhart [27] and Bratton and Kennedy [26].

### 2.5. Wavelet Analysis

Wavelet transformations provide useful decomposition of original time series by capturing useful information on various decomposition levels. Discrete wavelet transformation (DWT) is preferred in most of the forecasting problems because of its simplicity and ability to compute with less time. The DWT can be defined as

(6) $$\begin{array}{c} \psi_{m,n}\left(\frac{t-\tau }{s}\right)=\frac{1}{\sqrt{s_{0}^{m/2}}}\psi \left(\frac{t-n\tau_{0}s_{0}^{m}}{s_{0}^{m}}\right), \end{array}$$

where *m* and *n* are integers that control the scale and time. The most common choices for the parameters *s*_0_ = 2 and *τ*_0_ = 1. *ψ*(*t*) called the mother wavelet can be defined as ∫_−*∞*_^*∞*^ *ψ*(*t*)*dt* = 0.

For a discrete time series *x*(*t*) where *x*(*t*) occurs at discrete time *t*, the DWT becomes

(7) $$\begin{array}{c} W_{m,n}=2^{-m/2}\overset{N-1}{\underset{t=0}{\sum }}\psi \left(2^{-m}t-n\right)x\left(t\right), \end{array}$$

where *W*_*m*,*n*_ is the wavelet coefficient for the discrete wavelet at scale *s* = 2^*m*^ and *τ* = 2^*m*^ *n*. According to Mallat's theory, the original discrete time series *x*(*t*) can be decomposed into a series of linearity independent approximation and detail signals by using the inverse DWT. The inverse DWT is given by (Mallat [28])

(8) $$\begin{array}{c} x\left(t\right)=T+\overset{M}{\underset{m=1}{\sum }}\overset{2^{M-m-1}}{\underset{t=0}{\sum }}W_{m,n}2^{-m/2}\psi \left(2^{-m}t-n\right) \end{array}$$

or in a simple format as

(9) $$\begin{array}{c} x\left(t\right)=A_{M}\left(t\right)+\overset{M}{\underset{m=1}{\sum }}D_{m}\left(t\right), \end{array}$$

where *A*_*M*_(*t*) is called approximation subseries or residual term at levels *M* and *D*_*m*_(*t*)  (*m* = 1,2,…, *M*) are detail subseries which can capture small features of interpretational value in the data.

### 2.6. Principal Component Analysis

In an MLR, one of main tasks is to determine the model input variables that affect the output variables significantly. The choice of input variables is generally based on a priori knowledge of causal variables, inspections of time series plots, and statistical analysis of potential inputs and outputs. PCA is a technique widely used for reducing the number of input variables when we have huge volume of information and we want to have a better interpretation of variables (Çamdevýren et al. [29]).

The PCA approach introduces a few combinations for model input in comparison with the trial and error process. Given a set of centred input vectors *x*_1_, *x*_2_,…, *x*_*m*_ and ∑_*t*=1_^*m*^ *x*_*t*_ = 0, usually *n* < *m*. Then the covariance matrix of vector is given by

(10) $$\begin{array}{c} C=\frac{1}{l}\overset{l}{\underset{t=1}{\sum }}x_{t}x_{t}^{T}. \end{array}$$

The principal components (PCs) are computed by solving the eigenvalue problem of covariance matrix *C*,

(11) $$\begin{array}{c} \lambda_{i}u_{i}=Cu_{i},\;i=1,2,\ldots ,m, \end{array}$$

where *λ*_*i*_ is one of the eigenvalues of *C* and *u*_*i*_ is the corresponding eigenvector. Based on the estimated *u*_*i*_, the components of *z*_*t*_(*i*) are then calculated as the orthogonal transforms of *x*_*t*_:

(12) $$\begin{array}{c} z_{t}\left(i\right)=u_{i}^{T}x_{t},\;i=1,2,\ldots ,m. \end{array}$$

The new components, *z*_*i*_(*t*), are called principal components. By using only the first several eigenvectors sorted in descending order of the eigenvalues, the number of principal components in *z*_*t*_ can be reduced. So PCA has the dimensional reduction characteristic. The principal components of PCA have the following properties: *z*_*t*_(*i*) are linear combinations of the original variables, uncorrelated and have sequentially maximum variances (Jolliffe [30]). The calculation variance contribution rate is

(13) $$\begin{array}{c} V_{i}=\frac{\lambda_{i}}{\sum_{i=1}^{m}\lambda_{i}}\times 100\%. \end{array}$$

The cumulative variance contribution rate is

(14) $$\begin{array}{c} V\left(p\right)=\overset{p}{\underset{i=1}{\sum }}V_{i}. \end{array}$$

The number of the selected principal components is based on the cumulative variance contribution rate, which as a rule is over 85~90.

## 3. Computer Simulation

### 3.1. An Application

**Figure 2:**
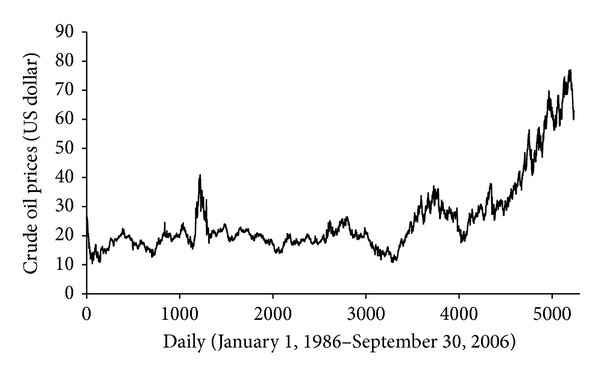
Daily crude oil prices from January 1, 198, to September 30, 2006.

In this study, the West Texas Intermediate (WTI) crude oil price series was chosen as experimental sample. The main reason of selecting the WTI crude oil is that these crude oil prices are the most famous benchmark prices, which are widely used as the basis of many crude oil price formulae. The daily data from January 1, 1986, to September 30, 2006, excluding public holidays, with a total of 5237 was employed as experimental data. For convenience of WMLR modeling, the data from January 1, 1986, to December 31, 2000, is used for the training set (3800 observations), and the remainder is used as the testing set (1437 observations).  Figure 2 shows the daily crude oil prices from January 1, 1986, to September 30.

In practice, short-term forecasting results are more useful as they provide timely information for the correction of forecasting value. In this study, three main performance criteria are used to evaluate the accuracy of the models. These criteria are mean absolute error (MAE), root mean squared error (RMSE), and *D*_stat_. The MAE and RMSE can be defined by

(15) $$\begin{array}{c} \text{RMSE}=\sqrt{\frac{1}{n}\overset{n}{\underset{t=1}{\sum }}{\left(y_{t}-{\hat{y}}_{t}\right)}^{2}},\\ \text{MAE}=\frac{1}{n}\overset{n}{\underset{t=1}{\sum }}\left|y_{t}-{\hat{y}}_{t}\right|. \end{array}$$

In crude oil price forecasting, improved decisions usually depend on correct forecasting of directions, of actual price, *y*_*t*_ and forecasted price, ${\hat{y}}_{t}$. The ability to predict movement direction can be measured by a directional statistic (*D*_stat_) (Yu et al., [1]), which can be expressed as

(16) $$\begin{array}{c} D_{\text{stat}}=\frac{1}{N}\overset{n}{\underset{t=1}{\sum }}a_{t}\times 100\%,\\ a_{t}=\{\begin{array}{cc} 1, & \text{if}\;\left(y_{t+1}-y_{t}\right)\left(\hat{y}{}_{t+1}-{\hat{y}}_{t}\right)\geq 0\\ 0, & \text{otherwise}. \end{array} \end{array}$$

### 3.2. Application and Result

At first, the MLR model without data preprocessing was used to model daily oil prices. In the next step, the preprocessed data which uses subtime series components obtained using discrete wavelet transform (DWT) on original data were entered to the MLR model in order to improve the model accuracy. For the MLR model, the original log return time series are decomposed into a certain number of subtime series components. Deciding the optimal decomposition level of the time series data in wavelet analysis plays an important role in preserving the information and reducing the distortion of the datasets. However, there is no existing theory to tell how many decomposition levels are needed for any time series.

**Figure 3:**
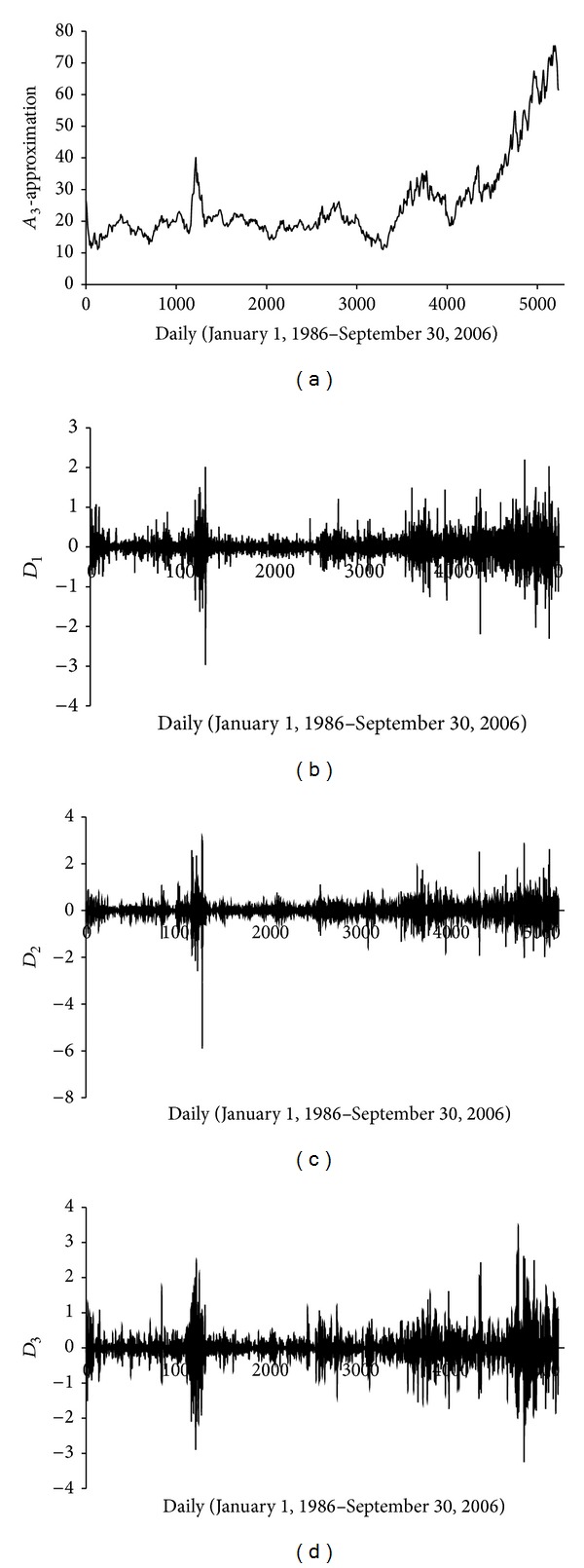
Decomposed wavelet subtime series components (Ds) of WTI crude oil price data.

In the present study, the previous log return of daily oil price time series is decomposed into various subtime series (DWs) at different decomposition levels by using DWT to estimate current price value. Three decomposition levels (2, 4, and 8 months) were considered for this study. For the WTI series data, time series of 2-day mode (DW1), 4-day mode (DW2) and 8-day mode (DW3), and approximate mode are presented in Figure 3.

For the WTI series, six input combinations based on previous log return of daily oil prices are evaluated to estimate current prices value. The input combinations evaluated in the study are (i) *r*_*t*−1_, (ii) *r*_*t*−1_, *r*_*t*−2_, (iii) *r*_*t*−1_, *r*_*t*−2_, *r*_*t*−3_, (iv) *r*_*t*−1_, *r*_*t*−2_, *r*_*t*−3_, *r*_*t*−4_, (v) *r*_*t*−1_, *r*_*t*−2_, *r*_*t*−3_, *r*_*t*−4_, *r*_*t*−5_, and (vi) *r*_*t*−1_, *r*_*t*−2_, *r*_*t*−3_, *r*_*t*−4_, *r*_*t*−5_, *r*_*t*−6_. In all cases, the output is the log return of current oil prices, *r*_*t*_.

Each of DWs series plays distinct role in original time series and has different effects on the original prices oil series. The selection of dominant DWs as inputs of MLR model becomes important and effective on the output data and has positive effect excessively on model's ability. The model becomes exponentially more complex as the number of subtime series as input variables increases. Using a large number of input variables should be avoided to save time and calculation effort. Therefore, the effectiveness of new series obtained by PCA is used as input to the MLR model. The PCA approach helps us to reduce the number of original variables to a set of new variables. Generally, the objective of PCA is to identify a new set of variables such that each variable, called a principal component, is a linear combination of the original variables. The new set of variables accounts for 85%−90% of the total variation were considered as the number of new variables.

**Figure 4:**
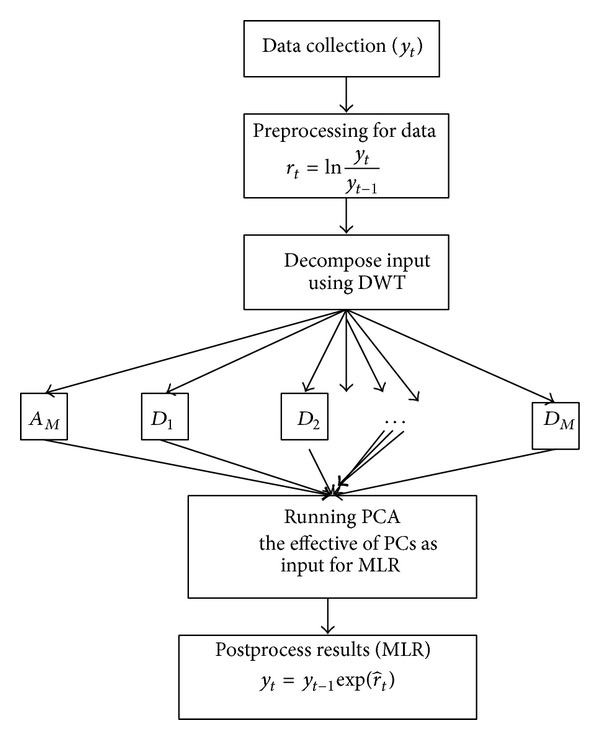
The structure of the WMLR model.

For example, taking two previous daily oil prices as a random variable. Every previous daily oil price time series are decomposed using DWT into three decomposition levels, respectively. Thus there were 8 subseries considered for the PCA analysis. The result of PCA analysis is shown in Table 1. Table 1 shows that the first four principle components can explain 84% variation of the data variation with the eigenvalues greater than 1 to be retained, in which all the 4 PCs were included in the MLR model. Thus the 8 original variables can be replaced by 4 new irrelevant variables. For training MLR, the PSO algorithm solving the recognition problem is implemented and the program code including wavelet toolbox was written in MATLAB language. The WMLR model structure developed in present study is shown in Figure 4.

The forecasting performances of the MLR and WMLR models in terms of the MAE, RMSE, and *D*_stat_ testing phase are compared and shown in Table 2. Table 2 shows MLR model; the M1 with 1 lag obtained the best MAE statistics of 0.6948 and the M6 with 6 lags obtained the best RMSE statistics of 0.9450, while the M1 with 5 lags obtained the best *D*_stat_ statistics of 0.4878. For WMLR, model M4 with 4 lags obtained the best MAE, RMSE, and *D*_stat_ statistics of 0.4834, 0.6572, and 0.6722, respectively. The equations of MLR with six input variables and WMLR with four input variables, respectively, are

(17) y^t=−0.025yt−1−0.047yt−2+0.22yt−3−0.082yt−4−0.060yt−5+0.0004yt−6,rt=0.076z1(t)−0.221z2(t)−0.213z3(t)−0.510z4(t)+0.416z5(t)−0.930z6(t),

where *z*_*i*_(*t*) are called principal components and ${\hat{y}}_{t}=y_{t-1}\exp(r_{t})$.

For further analysis, the best performance of the LR, WMLR, ARIMA, and ARIMA-GARCH models was compared with the best results of ARIMA and forward neural network (FNN) studied by Yu et al. [1]. In Table 3, it shows that WMLR outperform MLR, ARIMA, GARCH, Yu' ARIMA and Yu' FNN models in terms of RMSE statistics. This results show that the new series (DWT) have significant extremely positive effect on MLR model results.

**Figure 5:**
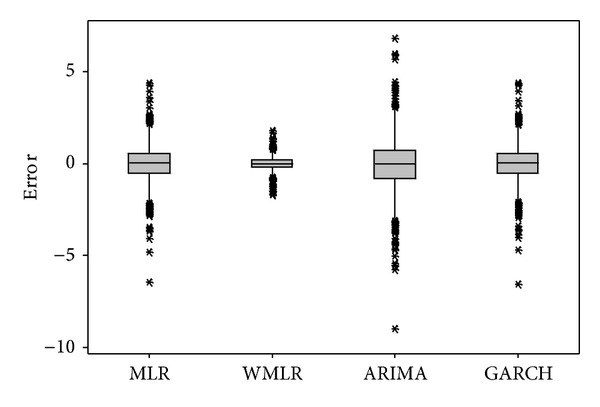
The errors of MLR, WMLR, ARIMA, and GARCH models for crude oil price forecasting.

Figure 5 shows the Box-plot for the ARIMA, ARIMA-GARCH, MLR, and WMLR models for testing period. It can be seen that the errors of WMLR model are quite close to the zero. Overall, it can be concluded that the WMLR model provided more accurate forecasting results than the other models for crude oil forecasting.

## 4. Conclusions

The accuracy of the wavelet multiple linear regression (WMLR) technique in the forecasting daily crude oil has been investigated in this study. The PCA is used to choose the principle component scores of the selected inputs which were used as independent variables in the MLR model and the particle swarm optimization (PSO) is used to adopt the optimal parameters of the MLR model. The performance of the proposed WMLR model was compared to regular LR, ARIMA, and GARCH model for crude oil forecasting. Comparison results indicated that the WMLR model was substantially more accurate than the other models. The study concludes that the forecasting abilities of the MLR model are found to be improved when the wavelet transformation technique is adopted for the data preprocessing. The decomposed periodic components obtained from the DWT technique are found to be most effective in yielding accurate forecast when used as inputs in the MLR model. The accurate forecasting results indicate that WMLR model provides a superior alternative to other models and a potentially very useful new method for crude oil forecasting. The WMLR model presented in this study is a simple explicit mathematical formulation. The WMLR model is much simpler in contrast to ANN model and can be successfully used in modeling short-term crude oil price. In the present study, three resolution levels were employed for decomposing crude oil time series. If more resolution levels were used, the results from WMLR model may turn out better. This may be a subject of another study.

## Figures and Tables

**Table 1:** Eigen value and cumulative variance contribution rate of the 8 principal components.

PC	1	2	3	4	5	6	7	8
Eigen value	1.97	1.79	1.59	1.33	0.67	0.41	0.21	0.03
Cumulative Variance Rate	0.25	0.47	0.67	0.84	0.92	0.97	1.00	1.00

**Table 2:** Forecasting performance indices of MLR and WLR.

Model Input	Lag	MLR			WMLR		
		MAE	RMSE	*D*_stat_	MAE	RMSE	*D*_stat_
M1	1	**0.6948**	0.9514	0.4788	0.6660	0.9001	0.5198
M2	1, 2	0.6972	0.9517	0.4781	0.6448	0.8842	0.5003
M3	1, 2, 3	0.6985	0.9545	0.4816	0.5345	0.7505	0.5797
M4	1, 2, 3, 4	0.6979	0.9550	0.4753	**0.4834**	**0.6572**	**0.6722**
M5	1, 2, 3, 4, 5	0.6976	0.9545	**0.4878**	0.5770	0.8046	0.5734
M6	1, 2, 3, 4, 5, 6	0.6969	**0.9450**	0.4850	0.5385	0.7389	0.6444

**Table 3:** The RMSE and MAE comparisons for different models.

Model	RMSE	MAE
ARIMA (2, 1, 5)	1.3835	1.0207

GARCH (1, 1)	0.9513	0.6947
MLR	0.9450	0.6969
WMLR	**0.6572**	**0.4834**
Yu' ARIMA (Yu et al., [1])	2.0350	—
Yu' FNN (Yu et al., [1])	0.8410	—
